# Depression, Anxiety, and Cigarette Smoking Among Patients with Tuberculosis

**DOI:** 10.1177/10547738221132096

**Published:** 2022-10-26

**Authors:** Alexandria Jones-Patten, Qiao Wang, Keneilwe Molebatsi, Thomas E. Novotny, Kamran Siddiqi, Chawangwa Modongo, Nicola M. Zetola, Bontle Mbongwe, Sanghyuk S. Shin

**Affiliations:** 1 University of California Irvine, CA, USA; 2 University of California Los Angeles, CA, USA; 3 University of Botswana, Gaborone, Botswana; 4 San Diego State University, CA, USA; 5 University of York, UK; 6 Victus Global Botswana Organisation, Gaborone, Botswana

**Keywords:** mental health, cigarette smoking, tuberculosis

## Abstract

Smoking adversely affects tuberculosis (TB) outcomes and may be associated with depression and anxiety among people diagnosed with TB in Botswana. We conducted a cross-sectional study among patients newly diagnosed with TB in Gaborone, Botswana, evaluating factors associated with self-reported cigarette smoking. We performed Poisson regression analyses with robust variance to examine whether depressive and anxiety symptoms were associated with smoking. Among 180 participants with TB enrolled from primary health clinics, depressive symptoms were reported in 47 (26.1%) participants and anxiety symptoms were reported in 85 (47.2%) participants. Overall, 45 (25.0%) participants reported current smoking. Depressive symptoms were associated with a higher prevalence of smoking (adjusted prevalence ratio [aPR]: 2.04; 95% confidence interval [CI]: 1.29–3.25) in the adjusted analysis. The association between anxiety symptoms and smoking did not reach statistical significance (aPR: 1.26; 95% CI: 0.77–2.05). Future studies should further investigate these associations when addressing TB care.

## Introduction

An estimated 1.5 million people died from tuberculosis (TB) globally in 2020; it is the 13th leading cause of global causes of death worldwide (WHO, 2021), and is the 3rd leading cause of death among communicable, maternal, neonatal, and nutritional diseases in Botswana (UNOPS & U. N. O. f. P. S., 2021). Tobacco smoking is associated with an increased risk of TB infection and TB disease (Lin et al., 2007). Research also suggests that smoking is associated with more extensive lung disease at the time of TB diagnosis (Leung et al., 2015), higher rates of TB infection (Alavi-Naini et al., 2012), and higher rates of TB treatment failures (Mahishale et al., 2015). Despite evidence showing these harmful links between smoking and TB, the WHO estimates that 1.3 billion people use tobacco globally, and more than 80% of smokers live in low- and middle-income countries (WHO, 2022).

### Depression, Anxiety, and Smoking, Generally Speaking

Compared with the general population, studies from different settings have consistently reported higher prevalence of common mental health disorders in patients diagnosed with TB (Duko et al., 2015; Hou et al., 2017; Kumar et al., 2016). In addition, in low- and middle-income countries, depression is more common among those diagnosed with TB compared to those without TB (Koyanagi et al., 2017), while literature examining anxiety in patients diagnosed with TB is limited. Several studies suggest an association between depression and smoking (Harris et al., 2019; Ojo-Fati et al., 2016; Stanton et al., 2020) and between anxiety and smoking (Stanton et al., 2020; Zvolensky et al., 2017); however, little is known about the association between anxiety and depression with smoking among people diagnosed with TB.

### TB and HIV co-infection

HIV is the single largest risk factor for TB, and TB is the leading cause of death among people living with HIV (Jackson-Morris et al., 2015). Among all incident TB cases, 8% of those cases were among people living with HIV globally (WHO, 2021), with HIV prevalence as high as 66% in high HIV-prevalent countries such as Botswana (Zetola et al., 2021). People living with HIV also have higher levels of depression/anxiety (Remien et al., 2019), which may lead to a need to find ways to cope. While the incidence of smoking is suggested to be higher in people living with HIV compared to the general population (Frazier et al., 2018), little is known about smoking and HIV among people with TB. In high HIV-prevalent settings, HIV status could be a confounder of the relationship between depression and anxiety with smoking among people living with TB.

In Botswana, an upper middle-income country, approximately 12.9% (23.9% of men and 2.8% of women) of people currently smoke cigarettes (World Health Organization, 2018). Rates of both TB and TB-HIV co-infection appear to be declining in Botswana, a country which offers universal healthcare coverage, with most of the population (84%) living within 5 km of a health facility (UNICEF, 2020). It is important to understand the determinants of cigarette smoking among patients infected with TB, as tobacco use can impact TB risk. To our knowledge, the associations between depression and anxiety with cigarette smoking have not been studied in people diagnosed with TB in Botswana. This study explores the association between common mental health conditions and smoking among patients diagnosed with TB, including depressive and anxiety symptoms. We hypothesize that there will be an independent association between both depressive symptoms and current cigarette smoking and anxiety symptoms and current cigarette smoking.

## Methods

### Study Design, Population, and Site Description

In an effort to explore the association between depressive symptoms, anxiety symptoms, and cigarette smoking in patients diagnosed with TB, this cross-sectional study was conducted from January to December 2019 at 12 primary healthcare facilities in Botswana’s largest city and capital, Gaborone. Participants were eligible for inclusion if they were at least 18 years of age and older, with a microbiologically confirmed TB diagnosis, and were recruited by our research staff at the time of receiving TB diagnosis. All patients diagnosed with TB at the participating facilities were consecutively enrolled for this study. All patients diagnosed with TB, regardless of disease site and drug susceptibility status, were eligible. All study procedures involving research participants were approved by the institutional review board at University of California, Irvine and Botswana Ministry of Health Human Research Development Committee, which provides research ethics oversight for public facilities. Informed consent was obtained by the collection of written signatures from all individual participants involved in the study.

#### Measures

The primary outcome variable was smoking status. For the analysis of smoking habits, participants were asked, “Are you a current smoker?” Current smoking was self-reported by participants, in which case participants were categorized into two groups: smokers and non-smokers. Participants who self-reported being a current smoker were then asked how many cigarettes they smoked daily. The independent variables were depression and anxiety.

Depressive symptoms were assessed using the Patient Health Questionnaire-9 (PHQ-9). This nine-item instrument analyzes the frequency of depressive symptoms up to 2 weeks prior to taking the questionnaire, using a 4-point Likert scale. Scores of 10 or higher indicate depressive symptoms (Kroenke et al., 2001). The internal reliability of the PHQ-9 in the current study was acceptable, with a Cronbach’s alpha of .74. This instrument has been validated in primary care clinic settings (Kroenke et al., 2001).

The Zung Self-Rating Anxiety Scale is a 20-item self-rating instrument which assesses cognitive, autonomic, motor, and central nervous system symptoms of anxiety using a 4-point Likert-type scale, ranging from “a little of the time,” “some of the time,” “good part of the time,” and “most of the time” (Zung, 1971). Scores of 36 or higher on the Zung scale were indicative of anxiety symptoms. The Zung scale demonstrated acceptable internal reliability with a Cronbach’s alpha of .80 in this study.

We also administered a questionnaire to collect data on age, sex, income, education, employment status, history of alcohol use, and smoking status. Clinical information collected included TB diagnosis, TB symptoms, and their duration. Rifampin-resistant results from Xpert MTB/RIF were available for some participants.

#### Data collection

Potential participants were identified first from the laboratory and clinic register and were approached for recruitment by Research Assistants, who then screened them for eligibility and obtained informed consent. The Research Assistants read consent materials in English and/or Setswana (official languages in Botswana) to patients who were illiterate. During consent, Research Assistants would take brief pauses to ask participants if they understood the information provided before continuing. Those who expressed they did not understand any part of the consent were provided more simplified information before proceeding with the remainder of the consent form and study enrollment. All participants verified their consent to participate by signature. Data on socioeconomic, demographic characteristics, and clinical information were collected in one-time interviews using the standardized questionnaires described above, prepared in both English and Setswana. Questionnaires were translated by a professional translation service in Botswana and then reviewed by a psychologist native to Botswana. The questionnaires were then field-tested on staff members of the research organization who were not involved in this research.

Participants whose total depressive symptom score corresponded with mild depression (score 5–9) were given a contact sheet for psychiatric services to seek help if symptoms worsened or persisted. Research assistants helped make appointments for participants scoring a 10 or higher on the PHQ-9 or those presenting with suicidal ideation.

#### Statistical analysis

We investigated whether depressive symptoms or anxiety symptoms were independently associated with the primary outcome of interest, smoking status, in people diagnosed with TB. We also dichotomized participants into having “no monthly income” and “some income,” and having “no education” and “some education,” as the variation between these categories was minimal. We examined the bivariate associations of current smoking with age, education, sex, monthly income, HIV status, anxiety scores, and depression scores. Poisson regression modeling with robust variance was used to estimate prevalence ratios (PRs) and 95% confidence intervals (95% CIs) for bivariate and multivariable analyses with current smoking status specified as the outcome variable (Greenland, 2004; Zou, 2004). The multivariate Poisson regression model assessed the association between mental health disorders and current smoking status adjusting for sex, income, and HIV status. We did not include education and age in the multivariable model since they did not improve our model fit and we had a relatively small sample size. A sensitivity analysis was also conducted; the analysis was restricted to men only as there were very few women identifying as smokers. We also conducted a sensitivity analysis to assess whether alcohol consumption would confound our results. We checked for multicollinearity by calculating the variance inflation factor in the final adjusted model and found no evidence of multicollinearity. Data were analyzed using SAS version 9.4 (SAS Institute, Cary, NC, USA).

## Results

A total of 180 participants were included in the study; the majority were males (64.4%, *n* = 116), and the mean age was 37 years, with an age range of 18–76 years. Of the 180 participants, 123 participants (68.3%) had a secondary education or less, while 40 participants (22.2%) had either tertiary education or certificate, diploma, or a college degree. Among all enrollees, depression was reported in 47 (26.1%) participants, while anxiety was reported in 85 (47.2%) participants. One-quarter (*n* = 45) of participants identified as smokers. Smokers were more likely to be males (91.1%, *n* = 41 vs. female: 8.9%, *n* = 4), and smoked a median of 10 cigarettes per day (67.0%, *n* = 30), while smokers reported smoking between 1 and 40 cigarettes daily. Comparing current smokers and non-smokers, current smokers were more likely to report depressive symptoms (PHQ-9 ≥10: 40.0% vs. 21.5%) and anxiety symptoms (Zung ≥36: 55.6% vs. 44.4%) (Table 1).

**Table 1.** Characteristics of Study Participants in Gaborone, Botswana, 2019 (*n* = 180).

Characteristics	Non-smoker *n*/*N*(%) or median (IQR)	Smoker *n*/*N*(%) or median (IQR)
Overall	135/180 (75.0)	45/180 (25.0)
Age (in years)	36.0 (27.0–46.0)	39.0 (30.0–43.0)
Number of cigarettes smoked per day	NA	10.0 (5.0–20.0)
HIV		
Negative	60/135 (44.4)	21/45 (46.7)
Positive	75/135 (55.6)	24/45 (53.3)
Depression		
No	106/135 (78.5)	27/45 (60.0)
Yes	29/135 (21.5)	18/45 (40.0)
Anxiety		
No	75/135 (55.6)	20/45 (44.4)
Yes	60/135 (44.4)	25/45 (55.6)
Education		
No	12/135 (8.9)	5/45 (11.1)
Yes	123/135 (91.1)	40/45 (88.9)
Income		
No	55/135 (40.7)	10/45 (22.2)
Yes	80/135 (59.3)	35/45 (77.8)
Gender		
Male	75/135 (55.6)	41/45 (91.1)
Female	60/135 (44.4)	4/45 (8.9)
Alcohol		
No	101/135 (74.8)	0
Yes	34/135 (25.2)	45/45 (100.0)
CD4* (cells/mm^3^)		
≤200	23/75 (30.7)	7/24 (29.2)
>200	40/75 (53.3)	15/24 (62.5)
Missing	12/75 (16.0)	2/24 (8.3)
ART*		
Never taken ART	25/75 (33.3)	6/24 (25.0)
Taking ART	45/75 (60.0)	14/24 (58.3)
Took ART but stopped	5/75 (6.7)	4/24 (16.7)

ART = antiretroviral therapy; IQR = interquartile range.

* Among HIV-positive patients only (*n* = 99).

In the unadjusted model, we found male participants were more likely than female participants to be current smokers (crude prevalence ratio [cPR]: 5.66; 95% CI: 2.12–15.07). Depressive symptoms (cPR:1.89; 95% CI: 1.15–3.10) and income (cPR: 1.98; 95% CI: 1.05–3.73) were also associated with current smoking (Table 2). After adjusting for HIV co-infection, income, and anxiety symptoms, depressive symptoms (adjusted prevalence ratio [aPR]: 2.04, 95% CI: 1.29–3.25) and male sex (aPR: 5.68, 95% CI: 2.09–15.43) remained to be significantly associated with current smoking (Table 3). In our sensitivity analyses, we found similar results for male participants only, (aPR: 2.22, 95% CI: 1.41–3.48), and a reduced level of association among alcohol consumers only (aPR: 1.44, 95% CI: 1.00–2.07) in the association between depressive symptoms and smoking (results not shown). We did not find a statistically significant association between anxiety symptoms and current smoking (aPR: 1.26; 95% CI: 0.77–2.05).

**Table 2.** Correlates of Current Smoking (*n* = 180)—Unadjusted Analysis.

Characteristic	Crude PR (95% CI)	*p* Value
HIV		
Negative	1.00	
Positive	0.94 (0.56–1.55)	.795
Depression		
No	1.00	
Yes	**1.89 (1.15–3.10)**	**.012**
Anxiety		
No	1.00	
Yes	1.40 (0.84–2.33)	.199
Education		
No	1.00	
Yes	0.83 (0.38–1.83)	.651
Income		
No	1.00	
Yes	**1.98 (1.05–3.73)**	**.035**
Gender		
Female	1.00	
Male	**5.65 (2.12–15.07)**	**.001**
Age	1.00 (0.99–1.02)	.630

CI = confidence interval; PR = prevalence ratio.

Bolded text indicates statistically significant results.

**Table 3.** Correlates of Current Smoking (*n* = 180)—Adjusted Analysis.

Characteristic	Adjusted PR (95% CI)	*p*-value
HIV		
Negative	1.00	
Positive	0.91 (0.57, 1.45)	.681
Depression		
No	1.00	
Yes	**2.04 (1.29–3.25)**	**.002**
Anxiety		
No	1.00	
Yes	1.26 (0.77–2.05)	.362
Income		
No	1.00	
Yes	1.55 (0.86–2.78)	.143
Gender		
Female	1.00	
Male	**5.68 (2.09–15.43)**	**.001**

CI = confidence interval; PR = prevalence ratio.

Bolded text indicates statistically significant results.

## Discussion

In this study, depressive symptoms were associated with current smoking among patients diagnosed with TB after adjusting for potential confounders. In addition, we found that men were more likely to engage in smoking than women in this study. This is consistent with data showing men in Botswana are more likely to smoke than women (Ritchie, 2019). Patients diagnosed with TB have reported both depressive symptoms (Rizvi, 2016; Shrestha et al., 2020) and smoking during treatment of TB (Altet et al., 2017; Leung et al., 2015), with both potentially leading to reduced treatment success. In addition to the hypothesis that this relationship could be bidirectional, another hypothesis for the association between depressive symptoms and smoking is the desire to self-medicate. Nicotine causes the release of dopamine; when smoking stops, depressed mood can occur as a result of nicotine withdrawal (Benowitz, 2009). The negative effect of depression may be enhanced among people diagnosed with TB, as treatment for TB can take a minimum of 6–9 months.

Anxiety did not demonstrate an association with current smoking in our sample. In a cross-sectional study among Chinese patients newly diagnosed with pulmonary TB, 18.37% (*n* = 1252) had significant anxiety symptoms, while current smokers had similar prevalence of anxiety symptoms as never smokers (aPR: 1.23; 95% CI: 0.79–1.91) (Wang et al., 2018). Elsewhere, a study, which explored psychological distress and smoking among patients diagnosed with TB in India, found anxiety rates to be higher at the beginning of treatment (24.4%) compared to 2-month follow-up (5.8%) and treatment completion (1.2%) (Febi et al., 2021). These results suggest that increased anxiety symptoms may be related to learning about new diagnoses. Consistent with the study by Wang et al., our study suggests there is no association between anxiety and smoking among patients diagnosed with TB. However, the sample size in our study may have been too small to detect an association. Longitudinal studies with a larger sample size should be conducted to further assess whether there is an association between anxiety and smoking in patients diagnosed with TB.

Our findings suggest that current efforts to help patients diagnosed with TB quit smoking may benefit from the management of multi-morbidity, including mental health and smoking cessation counseling. Smoking cessation advice and interventions are currently not routinely offered to patients in Botswana. Healthcare providers and service providers (e.g., social workers, community health workers, public health nurses) working directly with this population should attempt to explore why someone diagnosed with TB may be smoking, and monitor for behaviors and attitudes which mirror depression.

The findings of this study should be considered with several limitations. First, these data are from a cross-sectional study, leaving the direction of causality between smoking and depression unclear. However, other studies show consistent associations between depression and smoking (Ellis et al., 2015; Stubbs et al., 2018). Given the high proportion of smoking and depression among patients diagnosed with TB, a more consistent approach would be addressing the multi-morbidities of smoking and depression in TB treatment programs. Second, smoking status was assessed with a single question: “Are you a current smoker?” which is different than the questions used in many surveys; however, this question has been used to identify cigarette smokers in low- and middle-income countries (Giovino et al., 2012), where confirming 100 cigarettes smoked in one’s lifetime may impact smoking prevalence estimates (Levy et al., 2019). In addition, data about recent quit attempts were not collected. It is possible that people recently diagnosed with TB could have quit smoking upon learning of their TB diagnosis and were therefore classified as non-smokers. Furthermore, because we used a convenience sample in Botswana for this study, our results may not be generalizable to other TB populations. Next, there was not a formal validation of scale after translation from English to Setswana. Finally, data regarding smoking status, anxiety, and depression scores were collected by self-report, which may lead to misclassification of variables included in our model. However, research staff were trained to minimize this bias via objective interview techniques; for questions related to mental health disorders, participants were able to directly record their answers on the questionnaire.

## Conclusion

This study expands our understanding of the association between depression and smoking status among patients in Botswana. In addition, this study adds evidence to the need for specific public health efforts including smoking cessation interventions as part of TB treatment programs. Previous programs have evaluated smoking cessation as part of the core treatment program for people with TB (Siddiqi et al., 2013) and should be considered in future TB treatment programs. Mental health screenings should also be included in treatment of TB to provide person-centered care among these higher risk patients and further develop treatment plans for patients with TB multimorbidity. Future studies should employ larger sample size in different settings to validate our findings. In addition, future research should review the course of depression and anxiety and its association with smoking cessation during TB treatment (Siddiqi et al., 2021).
